# Large language models for structured reporting in radiology: past, present, and future

**DOI:** 10.1007/s00330-024-11107-6

**Published:** 2024-10-23

**Authors:** Felix Busch, Lena Hoffmann, Daniel Pinto dos Santos, Marcus R. Makowski, Luca Saba, Philipp Prucker, Martin Hadamitzky, Nassir Navab, Jakob Nikolas Kather, Daniel Truhn, Renato Cuocolo, Lisa C. Adams, Keno K. Bressem

**Affiliations:** 1 School of Medicine and Health, Department of Diagnostic and Interventional Radiology, Klinikum rechts der Isar, TUM University Hospital, Technical University of Munich, Munich, Germany; 2 Institute for Diagnostic and Interventional Radiology, Faculty of Medicine and University Hospital Cologne, University of Cologne, Cologne, Germany; 3 Institute of Diagnostic and Interventional Radiology, University Hospital of Frankfurt, Frankfurt, Germany; 4 Department of Radiology, Azienda Ospedaliero Universitaria (A.O.U.), Cagliari, Italy; 5 School of Medicine and Health, Institute for Cardiovascular Radiology and Nuclear Medicine, German Heart Center Munich, TUM University Hospital, Technical University of Munich, Munich, Germany; 6 Chair for Computer Aided Medical Procedures & Augmented Reality, TUM School of Computation, Information and Technology, Technical University of Munich, Munich, Germany; 7 Department of Medical Oncology, National Center for Tumor Diseases (NCT), Heidelberg University Hospital, Heidelberg, Germany; 8 Else Kroener Fresenius Center for Digital Health, Medical Faculty Carl Gustav Carus, Technical University Dresden, Dresden, Germany; 9 Department of Diagnostic and Interventional Radiology, University Hospital Aachen, Aachen, Germany; 10 Department of Medicine, Surgery and Dentistry, University of Salerno, Baronissi, Italy

**Keywords:** Artificial intelligence, Medical informatics, Natural language processing, Radiology, Electronic data processing

## Abstract

**Abstract:**

Structured reporting (SR) has long been a goal in radiology to standardize and improve the quality of radiology reports. Despite evidence that SR reduces errors, enhances comprehensiveness, and increases adherence to guidelines, its widespread adoption has been limited. Recently, large language models (LLMs) have emerged as a promising solution to automate and facilitate SR. Therefore, this narrative review aims to provide an overview of LLMs for SR in radiology and beyond. We found that the current literature on LLMs for SR is limited, comprising ten studies on the generative pre-trained transformer (GPT)-3.5 (*n* = 5) and/or GPT-4 (*n* = 8), while two studies additionally examined the performance of Perplexity and Bing Chat or IT5. All studies reported promising results and acknowledged the potential of LLMs for SR, with six out of ten studies demonstrating the feasibility of multilingual applications. Building upon these findings, we discuss limitations, regulatory challenges, and further applications of LLMs in radiology report processing, encompassing four main areas: documentation, translation and summarization, clinical evaluation, and data mining. In conclusion, this review underscores the transformative potential of LLMs to improve efficiency and accuracy in SR and radiology report processing.

**Key Points:**

***Question*** *How can LLMs help make SR in radiology more ubiquitous*?

***Findings*** *Current literature leveraging LLMs for SR is sparse but shows promising results, including the feasibility of multilingual applications*.

***Clinical relevance*** *LLMs have the potential to transform radiology report processing and enable the widespread adoption of SR. However, their future role in clinical practice depends on overcoming current limitations and regulatory challenges, including opaque algorithms and training data*.

## Introduction to structured reporting (SR)

One of the first known calls for standardization of radiologic report nomenclature was as early as 1922, published by Hickey et al for X-ray reports [1]. Over time, standardized and SR have become a persistent topic of discussion and analysis within the radiological community, with evolving perspectives on best practices and implementation strategies. Today, SR refers not only to the standardization of report content but also to the use of information technology (IT) tools for importing and organizing medical content that assists radiologists in creating reports [2]. While aiming to improve the quality, datafication/quantification, and accessibility of radiology reports, critics argue that structured reports can be overly rigid and potentially limit the radiologists’ ability to convey nuanced interpretations [3–5]. Additionally, SR templates require significant time and resources to create, maintain, and update, and the success of SR depends on the individual radiologists’ willingness to adopt the approach [3, 4, 6]. Despite these challenges, SR has gained increasing acceptance in the radiology community, with studies demonstrating its ability to reduce reporting errors, increase comprehensiveness, and improve compliance with national guidelines [7–9].

Throughout the history of SR, significant efforts have been made to standardize communication and reporting in radiology. In 1991, the American College of Radiology (ACR) published the first version of the “Guideline for Communication: Diagnostic Radiology” [10]. In 2006, RadLex®, a lexicon for radiology vocabulary, was introduced by the Radiological Society of North America (RSNA) with the goal of unifying the nomenclature used by radiologists [11]. In response to a consensus reached at the 2007 ACR Intersociety Conference, the RSNA SR Initiative created the RadReport Template Library, which includes text-based and XML-based report templates that link the RadLex® radiology vocabulary and other ontologies such as SNOMED CT® and LOINC® to corresponding report elements [7, 12, 13]. In 2018, the European Society of Radiology (ESR) published a white paper on SR in radiology, calling for international, intersociety, and industry collaboration to successfully implement SR in radiology practice at large [3]. This has recently been updated, noting that implementation in clinical routine is still lacking and that policymakers should incentivize the use of structured radiology reporting [14]. Moreover, the updated ESR statement highlights large language models (LLMs) as a potential solution for integrating SR into radiologists’ workflows [14].

Therefore, this narrative review aims to provide a detailed overview of the current literature on LLMs for SR in radiology. In addition, we capture limitations, regulatory challenges, and the evolution of natural language processing (NLP) and other applications of LLMs in radiology and beyond. SR was defined as “an IT-based method to import and arrange the medical content into the radiological report” to acknowledge the difference between structured and standardized reporting, according to Nobel et al [2]. Due to the lack of a universal definition of LLMs, we defined LLMs as any large-scale transformer-based generative language model that can follow instructions, e.g., through training with reinforcement learning from human feedback [15].

## The evolution of NLP: from SMLs to LLMs

Technically, the integration of picture archiving and communication systems and radiology information systems (RIS) with electronic health records could have been a milestone in facilitating the adoption of SR, enabling the implementation of structured templates, and sharing reports across different institutional or healthcare systems [16]. Still, manual efforts, such as selecting the appropriate template, extracting the relevant free text into the correct structured format field, or translation tasks, may have contributed to the reluctance to adopt SR in clinical practice. In addition, the use of SR templates can be time-consuming, as the user interfaces usually require a lot of interaction and thus may disrupt the workflow.

From a computer scientist’s perspective, NLP could be the solution to these problems. NLP is a subfield of computer science that focuses on enabling computers to understand, interpret, generate, and manipulate human language using linguistics and logic [17, 18]. However, NLP was not always as promising for aiding radiologists in SR as it now appears to be. Starting with the first statistical language models in the 1990s, the need to estimate probabilities for an exponentially growing number of word sequences, many of which may not even appear in the training data, led to data sparsity problems and made it difficult to accurately predict the probability of word sequences, especially in domains with rich and specialized vocabularies such as medicine [19–21]. In the 2000s, long short-term memory networks (LSTM) represented a breakthrough by using deep neural networks to process language [22, 23]. LSTMs, unlike their predecessors, possessed the ability to recognize and use longer-ranging patterns in text. They achieve this by constructing a word prediction mechanism that relies on cumulative contextual information, which equips them with the capability to address a wide range of common NLP tasks efficiently [21]. However, the sequential processing of LSTMs is slow and computationally intensive for longer texts. In 2018, the first transformers-based language models, such as BERT (bidirectional encoder representations from transformers), were introduced, able to learn more differentiated language representations and linguistic patterns using large datasets of text [24]. Leveraging self-attention mechanisms (a technique allowing models to focus on relevant parts of the input), these models achieved a deeper contextual understanding, while their inherent parallelizability enabled efficient large-scale training [21]. This allowed transformer models to leverage knowledge from massive amounts of training data and generalize to new tasks and data with little or no additional training. For healthcare tasks, transformers have also shown promising performance when fine-tuned to specific downstream tasks in the biomedical domain [25]. Eventually, the term large language model (LLM) was adopted to describe transformer-based models with billions of parameters that are trained on vast amounts of textual data, allowing them to learn more nuanced relationships and solve more NLP complex tasks [15, 26–28]. While LLM is usually used to refer to models that just process natural language, similar transformer-based architectures that can also process images are termed visual language models [29]. These advancements have significantly increased the accessibility and interest in leveraging LLMs for medical applications among healthcare professionals and the medical industry [30]. To date, LLMs may be the most capable models for automating SR in radiology. In this review, we focus on LLMs that process natural language input, such as free-text radiology reports, to generate structured reports. Models that directly analyze medical images to generate reports, while related, are beyond the scope of this review. Also, BERT approaches for SR were not included in the review, as they are based on encoder-only architectures and have already been discussed in detail [31].

## Review of current LLMs for SR

Two authors (F.B. and L.H.), in consensus with a third author (K.K.B.), independently searched the Web of Science, PubMed, and Embase databases using the syntax search strategy of Nobel et al (radiol* AND structur* AND report*) without date restriction as of March 9, 2024, identifying six articles on LLMs for SR in radiology [32]. During revision, one article was suggested by peer review, and an additional Google Scholar search by the authors at the time of revision led to the inclusion of three more studies.

Overall, we found that the current literature on LLMs for SR in radiology is limited, including ten articles, all of which investigated either the capabilities of generative pre-trained transformers (GPT)-3.5 (*n* = 2) [33, 34], GPT-4 (*n* = 5) [35–39], or both (*n* = 3) [40–42] for structured radiology reporting, while one study additionally examined the performance of Perplexity and Bing Chat (now Microsoft Copilot; uses the Microsoft Prometheus model, built upon OpenAI’s GPT-4) [41] and one other study IT5 (text-to-text transfer transformer fine-tuned for Italian language) [34]. The radiological domains studied were heterogeneous, including interventional radiology (*n* = 1) [35], whole-body computed tomography (CT) reports (*n* = 2) [40, 41], magnetic resonance imaging (MRI) and/or CT reports of various body regions (*n* = 3) [34, 36, 38], contrast-enhanced ultrasonography (CEUS) examinations of the liver (*n* = 1) [37], ultrasound reports of thyroid nodules (*n* = 1) [42], reports of distal radius fractures (*n* = 1) [33], and chest X-rays (*n* = 1) [39]. Most studies examined the capabilities of LLMs for the transformation of free text into structured reports (*n* = 5) [33–38, 40, 42], while one study evaluated the knowledge of LLMs about SR in radiology and their ability to generate examples of structured reports [41], one study additionally evaluated the automated text report generation [33], and one study developed a prompt-guided approach using anatomical region detection and feature extraction to generate SR [39]. Several studies investigated the multilingual application of LLMs in SR by either conducting the full analysis in Italian (*n* = 3) [34, 40, 41] or Chinese (*n* = 1) [37], including an initial translation task from Japanese (*n* = 1) [35], or additionally evaluating the performance on a benchmark with German chest radiography reports (*n* = 1) [36]. Due to the differences in study designs, including qualitative and quantitative methods, as well as the heterogeneous evaluation of the performance for SR, a meta-analysis was not feasible. However, all studies reported a positive outlook, with one study demonstrating 100% accuracy for GPT-4 in automatically matching MRI/CT reports of different body regions to the appropriate report template, converting the reports to JSON files, and structuring the report without errors, loss of accuracy, or indication of additional findings [36]. An overview of all studies included, with study information, methods, and results, is provided in Table 1, while Table 2 provides an overview of the models used and their specific features.

**Table 1:** Overview of the included studies, their characteristics, purpose, methods, results, and conclusions

Author, year, country, and study design	Purpose	Methods	Results	Main conclusions
1. Sasaki et al, 2024; Japan; quantitative nonrandomized [35]	To assess GPT-4’s capabilities in IR report structuring and translation from Japanese.	Two hundred and seventy-seven of 899 Japanese IR reports recorded in 2022 from a single center were randomly selected; GPT-4 was prompted to transform the Japanese free-text report into a structured JSON format in English; two physicians independently evaluated the original and structured reports in nine categories.	Mean age: 68 years; 188/89 males/females; 85% of IR reports were deemed appropriate in every category (median: 94%), except for the “plan” section, where 49% of reports were deemed appropriate, 27% of reports contained hallucinations, and 19% contained misinterpretations.	LLMs can create structured IR reports from free text and process languages other than English but require careful consideration and possibly additional information for each procedure and report template to define the structured format terminology.
2. Mallio et al, 2023; Italy; quantitative nonrandomized [40]	To examine the capabilities of GPT-3.5 and GPT-4 in transforming free-text radiological reports into structured formats, including word count reduction and quality of the structured report in Italian.	Sixty fictitious total-body CT reports, randomly divided into three groups of 20 reports, were created by consensus of one radiologist and one radiology resident; GPT 3.5 Turbo and GPT-4 were prompted for (1) detailed SR, (2) focus on essential information, and (3) only extract pathological elements; quantitative analysis using word count reduction and C-RADS.	Prompt 1: GPT-3.5/GPT-4: word count reduction: 23.6%/36.7%; GPT-3.5/GPT-4 C-RADS recall range: E4 minimum 79%/80 to E3 maximum 89%/93%; prompt 2: GPT-3.5/GPT-4: word count reduction: 49.9%/75%; GPT-3.5/GPT-4 C-RADS recall range: E2 minimum 65%/E1 59% to E3 maximum 77%/77%; prompt 3: GPT-3.5/GPT-4: word count reduction: 47%/73.2%; GPT-3.5/GPT-4 C-RADS recall range: E1 minimum 62%/36% to E4 maximum 90%/83%.	Both models demonstrated the ability to transform free-text radiology reports into a structured format and reduce verbosity. Some findings, even those of potential clinical importance, may be missed. GPT-4 demonstrated a greater ability to reduce the number of words in reports compared to GPT-3.5.
3. Mallio et al, 2024; Italy; qualitative [41]	To examine the knowledge of GPT-4, GPT 3.5, Perplexity, and Bing about SR in radiology and their ability to automatically create examples of structured radiology report templates of total-body CT in Italian.	Each model was asked three prompts: (1) Tell me about structured reports in radiology, (2) Tell me more about it, at least 2000 tokens; and (3) please provide me with an example of a structured report of a total-body CT examination; include as much detail as possible. The format must be tabular; with qualitative analysis of the output.	All models demonstrated good knowledge about SR, with examples provided. GPT-4 and Perplexity offered broad, detailed responses, whereas GPT 3.5 and Bing provided concise, targeted replies. GPT-4 provided comprehensive, detailed, and accurate answers, clearly explaining structured radiological reports, benefits, and tabular examples. Perplexity showed a comprehensive understanding of structured reports. GPT 3.5 delivered relevant information and templates, and Bing was the most concise yet still comprehensive and focused.	All models show promising potential in producing structured radiology reports. However, integrating human expertise and supervision is crucial for generating precise and comprehensive structured reports.
4. Adams et al, 2023; Germany, USA; quantitative nonrandomized [36]	To evaluate GPT-4’s ability to automate the conversion of free-text radiology reports into structured templates in English, as well as the performance in CXR classification in German.	One hundred seventy fictitious CT and MRI reports were created by two radiologists for various examinations and body regions; templates were chosen based on the RadReport Template Library, among others; evaluation of consistency and accuracy of generated reports by two radiologists; multilingual evaluation on the medBERT.de CXR classification benchmark involving 583 German CXR reports.	One hundred percent of reports successfully matched to the most appropriate report template and successfully transformed into JSON files without error, loss of accuracy, or indicating additional findings; GPT-4 outperformed the existing state-of-the-art model on the medBERT.de benchmark in detecting three of four pathological findings and one therapeutic device category.	GPT-4 is an effective and cost-effective tool for post hoc SR in radiology.
5. Bosbach et al, 2023; Germany, Poland, Switzerland, Hungary, Malaysia; quantitative nonrandomized [33]	To evaluate GPT-3.5’s capabilities in automated text report generation and structuring for distal radius fracture in English.	Nine fictional test cases of distal radius fracture were created; input information for the report followed the structure of RSNA templates and AO fracture classification; five iterations were performed for findings/impression separately and merged; evaluation using cosine similarity and a radiological score card for overall quality in five categories by three radiologists	Text similarity reached plateaus; “findings/impressions separate” category score card [strongly agree]: exam information: 97%, fracture findings: 93%, impressions suitable: 45%, grammar correct: 87%, style format: 100%; “only impression” category score card [strongly agree]: exam information: 72%, fracture findings: 84%, impressions suitable: 73%, grammar correct: 68%, style format 100%	GPT-3.5 produced high-quality reports and can adjust output files in response to minor changes in input command files. Shortcomings were found in technical terminology and medical interpretation of findings.
6. Wang et al, 2024; China; quantitative nonrandomized [37]	To compare the performance of doctors using conventional free-text reports with those employing structured reports generated by GPT-4 for CEUS liver examinations in Chinese.	One hundred fifty-nine CEUS reports from a single center between 2017 and 2023 of patients with suspected solid liver nodules; evaluation by 30 doctors with varying experience for accuracy and efficiency of either structured or original free-text reports; additional qualitative analysis of doctor’s responses.	Age range: 13–85 years, gender: 31%/69% female/male; quantitative analysis: significant improvements in diagnostic efficiency (20 min vs 17 min) and accuracy (73% vs 79%) for doctors using GPT-4-generated structured reports; qualitative analysis of generated reports: clarity and organization were rated good, areas for improvement were negative information, incomplete or insufficient details on specific features, and missing general patient clinical data.	GPT-4-generated structured reports enhance diagnostic efficiency and accuracy in medical imaging, specifically in liver nodule CEUS examinations.
7. Bergomi et al, 2024; Italy; quantitative nonrandomized [34]	To evaluate and compare IT5 and GPT-3.5 for automatically converting Italian free-text radiology reports into structured reports.	One hundred seventy-four free-text radiology CT reports of lymph node lesions from a single center; comparison of batch-truncation and ex-post combination strategies; evaluation using strict accuracy, F1, format accuracy, and rating on a 5-point Likert scale by two radiologists.	IT5 ex-post combination performed best: 51.7% strict accuracy, 77.4% F1 score, 95.4% format accuracy overall; 64.7% strict accuracy, 78.1% F1 for multichoice; 33.7% strict accuracy, 56.3% F1 for free text. GPT-3.5 scored lower (25.5% strict accuracy, 40.3% F1) but received higher human ratings (correctness: 3.5 ± 1.6, completeness: 3.81 ± 1.1) than IT5 (correctness: 2.51 ± 0.97, completeness: 2.43 ± 0.9).	Smaller fine-tuned models like IT5 can perform well in clinical information extraction for structured report filling. LLMs like GPT-3.5 produce more human-like responses.
8. Pan et al, 2024; China; quantitative nonrandomized [38]	To assess GPT-4’s capabilities in automating the transformation of free-text radiology reports into FHIR-compliant structured formats.	Selected ten representative cases out of 40 de-identified radiology reports from a single center; designed FHIR radiology report templates using LHC-Forms toolkit; used GPT-4 to convert free-text reports to FHIR-structured JSON format; evaluation by 15 radiology staff members using a 5-point Likert scale questionnaire for accuracy and completeness; calculation of Cronbach’s alpha for internal consistency.	All report types received average ratings above 4.5 out of 5 for accuracy and completeness; cranial CT reports received the highest ratings (4.9 for both accuracy and completeness); high Cronbach’s alpha values across all report types (ranging from 0.910 to 0.987); X-ray reports scored highest in subgroup analysis (4.86 for accuracy and 4.84 for completeness).	GPT-4 effectively transforms radiology reports into FHIR-compliant structured formats, slightly varying performance across different imaging modalities.
9. Jiang et al, 2024; China; quantitative nonrandomized [42]	To assess the accuracy and reproducibility of GPT-3.5 and 4 in generating structured thyroid ultrasound reports.	One hundred thirty-six free-text thyroid ultrasound reports (184 nodules) from a single center; template creation based on ACR-TIRADS guidelines; two radiologists evaluated reports for quality, nodule categorization accuracy, and management recommendations; evaluation using 5-point Likert scales and ICC for consistency.	Mean age: 52 years, 61/75 male/female; GPT-3.5 generated more satisfactory structured reports than GPT-4 (74.3% vs 25.4); GPT-4 outperformed GPT-3.5 in nodule categorization accuracy (69.3% vs 34.5%); GPT-4 provided more comprehensive/correct management recommendations (OR = 1.791, *p* < 0.001); GPT-4 showed higher consistency in categorizing nodules (ICC = 0.732 vs 0.429); both showed moderate consistency in management recommendations (ICC ≈ 0.55–0.57).	ChatGPT shows potential in transforming free-text thyroid ultrasound reports into structured formats; GPT-3.5 excels in generating structured reports, while GPT-4 is superior in nodule categorization and management recommendations.
10. Li et al, 2024; China; quantitative nonrandomized and qualitative [39]	To develop a prompt-guided approach using a pre-trained GPT-4 model to generate structured CXR reports, enhancing clinical interpretability and interactivity.	Faster R-CNN for anatomical region detection and feature extraction; generation of region-specific sentences and integration with clinical context prompts to produce structured reports; evaluation on the MIMIC-CXR dataset using BLEU, METEOR, ROUGE, and CE metrics.	BLEU-4: 0.131, METEOR: 0.161, ROUGE-L: 0.261; CE metrics: F1: 0.441, precision: 0.469, recall: 0.470. Structured reports demonstrated superior accuracy in capturing anatomical and clinical details compared to baseline models.	The proposed method can generate interpretable and interactive structured CXR reports. The anatomy-guided approach improves report structure and clinical relevance. The integration of clinical context prompts allows for physician input and enhances report customization.

*ACR TI-RADS* American College of Radiology Thyroid Imaging Reporting & Data System, *AO* arbeitsgemeinschaft für osteosynthesefragen, *CE* clinical efficiency, *CEUS* contrast-enhanced ultrasonography, *C-RADS* colonography reporting and data system, *CT* computed tomography, *CXR* chest X-ray, *EHR* electronic health record, *FHIR* fast healthcare interoperability resources, *GPT* generative pre-trained transformers, *ICC* intraclass correlation coefficient, *IR* interventional radiology, *IT5* text-to-text transfer transformer fine-tuned for Italian language, *LLMs* large language models, *MRI* magnetic resonance imaging, *OR* odds ratio, *R-CNN* region-based convolutional neural network, *RSNA* Radiological Society of North America

**Table 2:** Overview of the models used in the literature for SR and their characteristics

Model	URL	Parameter size (estimated)	Open-source	Multimodal use	Costs	Capabilities	Limitations	Prospects	Clinical practice status
GPT-3	https://platform.openai.com/docs/models/gpt-base	175b	No	No	API	Generating structured reports is feasible.	Less accurate than GPT-3.5 and GPT-4 for most metrics.	Release of new, more powerful OpenAI models expected.	Not approved
GPT-3.5	https://platform.openai.com/docs/models/gpt-base	20b	No	Yes	API	Generating structured reports is feasible.	Less accurate than GPT-4 for most metrics.		Not approved
GPT-4	https://platform.openai.com/docs/models/gpt-base	1.76t	No	Yes	Pro plan, API	Multilingual and multimodal capability, the highest accuracy of all OpenAI models.	May make mistakes, hallucinate, or miss information.		Not approved
Perplexity.ai	https://www.perplexity.ai	In-house models up to 70b	No	Yes	Pro plan, API	Generating structured reports is feasible.	Not reported	Release of new, more powerful in-house models expected.	Not approved
Microsoft Copilot (former Bing Chat)	https://copilot.microsoft.com/	Based on GPT-4	No	Yes	Pro plan	Generating structured reports is feasible.	Not reported	Depends on the development of new models by OpenAI.	Not approved
IT5	https://github.com/gsarti/it5	Up to 738 m	Yes	No	Free	The fine-tuned model performed well compared to GPT-3.5.	Limited to the Italian language, less human responses than GPT-3.5.	Smaller, more efficient open-source models of the T5 family could be used for language-specific SR.	Not approved

*API* application programming interface, *CXR* chest X-ray, *GPT* generative pre-trained transformers, *IT5* text-to-text transfer transformer fine-tuned for Italian language, *SR* structured reporting

Ultimately, commercial solutions leveraging LLMs for SR already exist on the market. For example, Munich-based startup Smart Reporting and the San Francisco-based company Rad AI deploy generative AI models via Amazon Bedrock for voice-guided and data-driven documentation and report structuring [43, 44]. However, as peer-reviewed study data and information about the exact architectures used are unavailable, However, due to the lack of peer-reviewed study data and information on the exact models used, these solutions could not be evaluated in this review.

### Limitations

Although there is great potential to apply LLMs for SR, the reviewed studies emphasized several limitations of LLMs tested, including hallucinations, i.e., the creation of entirely fictitious or false information that has no basis in the input provided or in reality, misinterpretation of (medical) information, shortcomings in technical terminology, varying performance depending on the task, and missing information [33, 35, 37, 40, 41].

Moreover, when looking at the broader literature on LLMs in medicine, several other limitations have been reported [45]. For example, safety issues were identified, with GPT-3.5 and GPT-4 incorrectly recommending advanced life support techniques in 13.6% of pediatric emergency cases [46]. Likewise, several studies have noted the provision of outdated medical information [47, 48]. Readability was another challenge, with GPT-3.5’s responses on uveitis being significantly above the recommended 6th-grade level for patient education materials [49]. While less frequently reported, biases were also observed, such as biases related to underserved racial groups in cardiovascular disease information [50]. Another increasingly recognized limitation of AI applications is the unexplainable nature of the decision-making process. Specifically, many authors argue that particularly in the medical domain, it is necessary to understand and agree with how an AI system reaches its conclusions in order to use it confidently and ethically in clinical decision-making [51–53]. However, balancing the high accuracy of complex models with the need for interpretability remains a significant challenge in medical AI applications [54].

To address the limitations of LLMs, several solutions have been proposed. For example, the integration of external knowledge bases or automated fact-checking systems aims to improve the accuracy of LLM outputs by verifying information against reliable medical databases [55]. However, these systems are only as reliable as the databases they query, and their integration can be complex and computationally intensive. Self-assessment methods (e.g., the dual role of GPT-4 in generating and scoring responses) have shown promise and could allow LLMs to not only produce structured reports but also independently access the quality of the report; however, this is currently challenged by overly rigorous assessments in free-form tasks [56]. Metrics like ROUGE-L, although useful for short-answer tasks, often fail in creative or open-ended scenarios due to their reliance on textual overlap [56]. Furthermore, while multilingual instruction tuning (e.g., BLOOMZ and M3IT) enhances cross-lingual capabilities, its effectiveness varies significantly across different languages and task types [56]. Domain-specific fine-tuning, which involves training LLMs on high-quality, peer-reviewed medical literature, can improve their understanding of medical terminology and concepts [57]. However, the process requires significant resources and time, and there’s always the risk that the model will still miss subtle nuances in medical texts. Human-in-the-loop approaches combine LLM capabilities with expert supervision to detect and correct errors [58]. While this hybrid approach can improve accuracy, it is labor-intensive and may not scale effectively for large datasets. In addition, it can introduce human bias, potentially undermining the objectivity that automation is intended to provide. Enhancing prompt engineering to guide LLMs toward more accurate and comprehensive responses is another proposed solution [59]. However, creating effective prompts requires deep expertise and constant adaptation, as prompts that work well in one context may fail in another. Finally, implementing retrieval-augmented generation allows LLMs to dynamically retrieve information from verified medical databases during the generation process [60]. This technique can ensure that models have access to the latest information, but it also increases the computational load and may suffer from the same reliability issues as the underlying databases.

### Regulatory challenges

Many countries are planning to introduce AI-related legal frameworks that AI providers must comply with. However, progress in introducing these frameworks varies greatly from region to region. In the United States, for example, federal authorities have not yet introduced specific safeguards for AI, and thus, AI medical products are still subject to the standard Food and Drug Administration approval process [61]. Likewise, the timeline for enacting Canada’s AI and Data Act, which was initially proposed in 2022, remains uncertain [62]. The Asia-Pacific region is currently witnessing a rapid development of AI regulatory frameworks with different approaches across jurisdictions. While most countries focus on high-level, principles-based guidelines and voluntary measures, a few are moving toward more specific AI regulations. In 2023, China introduced a framework for generative AI services, along with rules for deep synthesis, algorithmic recommendations, and ethical review [63]. South Korea and Taiwan are considering AI-specific legislation, although these are still in the draft stage [63]. Many other countries, including Australia, Japan, Singapore, India, Hong Kong, Thailand, and Vietnam, currently rely on non-binding guidelines and existing laws to address AI-related issues [63].

**Fig. 1:**
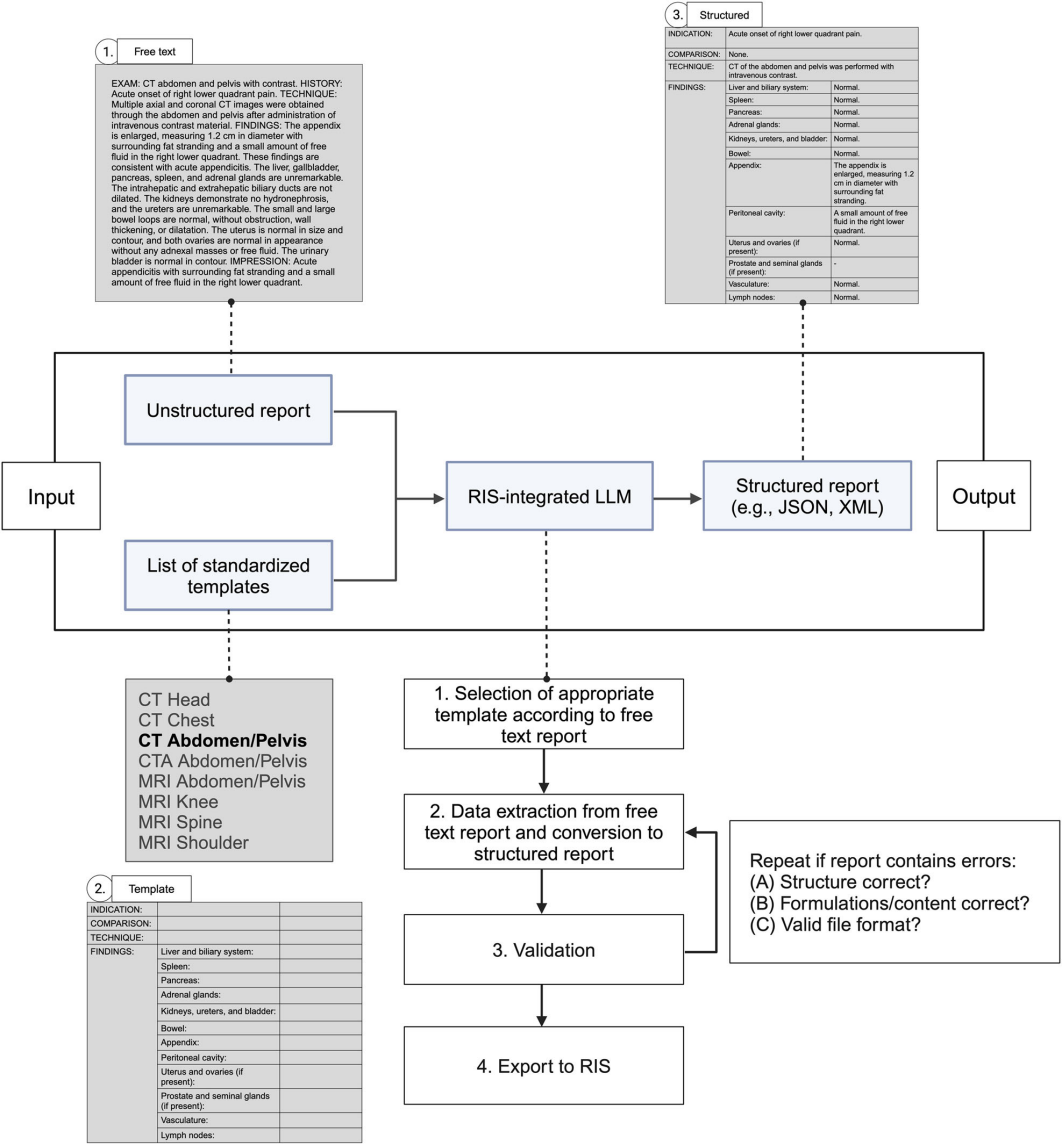
Workflow example for the integration of LLMs to structure radiology reports in clinical practice. RIS, radiology information systems. Created with BioRender.com

In Europe, on the other hand, the first legally binding framework for AI became effective with the adoption of the AI Act of the European Union (EU) on March 13, 2024 [64]. This applies not only to companies based in the EU, but also to all providers from third countries that want to place their solutions on the EU market, as well as is likely to serve as an orientation for other countries in regulating AI products. The AI Act employs a risk-based categorization for AI systems, including unacceptable, high, limited, and minimal risk, with varying regulatory requirements [65]. Within this framework, LLMs are identified as foundational models, meaning that they are AI systems trained on large datasets for broad applicability and adaptability to diverse tasks, including some for which they were not specifically developed and trained [66]. However, under the Act’s criteria, a foundational model only achieves a high-risk designation if it is part of a general-purpose AI system, a scenario that is unlikely for LLMs used in focused, controlled settings without autonomous decision-making, such as for SR in radiology [67]. We provide a schematic illustration of integrating LLMs with manual validation for generating SR outputs in the radiologist’s workflow in Fig. 1. Still, core requirements, such as a quality management system, data governance, risk mitigation, efficiency (energy and resource use), performance, predictability, interpretability, corrigibility, safety, cybersecurity, technical documentation, and EU database registration, must be met to comply with the EU law under the AI Act [65]. In addition, transparency requirements, legal content safeguards, and training data/copyright transparency are required for generative AI models such as ChatGPT. In fact, proprietary models like ChatGPT, which provide limited access and information about their underlying algorithms, training data, and data processing and storage mechanism, currently do not meet these requirements [68]. In contrast, publicly available open-source models can circumvent stricter requirements if their license allows access, use, modification, and distribution of the model and its parameters and if there is no association with high-risk or prohibited applications and no risk of manipulation [65]. Therefore, given the primary focus on proprietary models in our analyzed studies, future studies may assess and compare the capabilities of open-source medical models, such as BioMistral, for structuring radiology reports to potentially navigate these regulatory requirements more freely [69].

Thus, although current LLM applications may already have the capabilities to successfully automate SR, their future in clinical practice depends on overcoming their current limitations and regulatory challenges. As this field evolves, continued dialog between developers, medical professionals, legal experts, and regulators is critical to effectively overcome these challenges and successfully deploy LLMs for SR in radiology.

## Further applications for LLMs in radiology

**Fig. 2:**
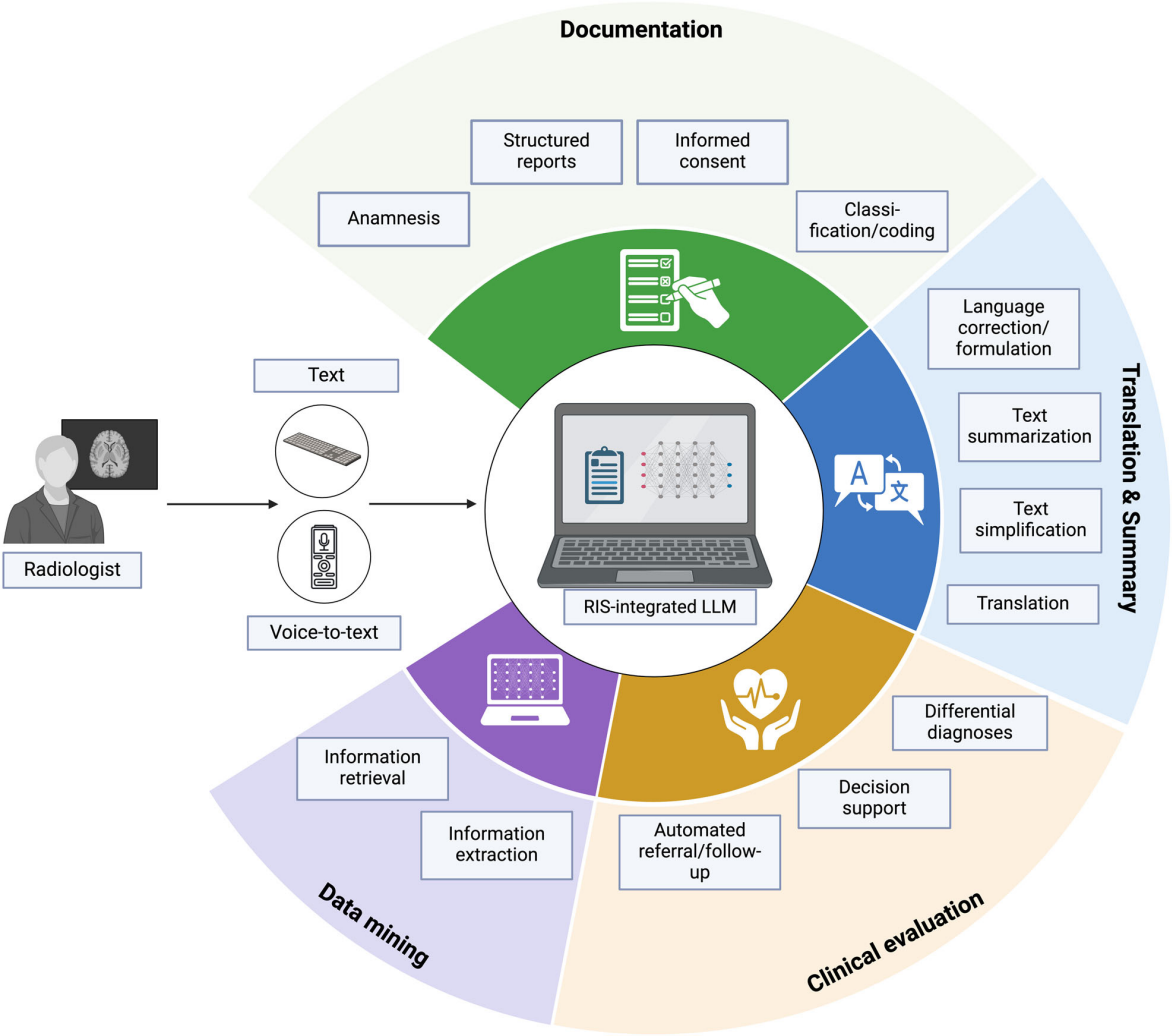
Schematic illustration of the present and future role of LLMs in the domain of radiology report processing, categorized across four main areas: documentation, translation and summarization, clinical evaluation, and data mining. RIS, radiology information systems. Created with BioRender.com

Looking beyond the horizon of SR, LLMs have the potential to solve various tasks in the radiology domain. In fact, the ability to automate the structuring and standardization of a wide range of medical documents—from individual patient records to entire institutional databases—offers enormous potential for data mining and harmonizing data across different healthcare systems [70]. Even before report generation, LLMs could enhance clinical documentation by taking and compiling a patient’s medical history prior to radiology exams, including automated patient triage, providing an accessible chatbot for patients, an initial overview for physicians, and facilitating the creation and documentation of consent forms [71–73]. In addition, LLMs could predict the disease or region of interest based on the information provided by the patient and automatically select the most appropriate imaging protocol, including the decision to use a contrast agent and the individual amount needed [74, 75]. During report creation, LLMs may be used to enhance the voice-to-text generation of reports by detecting speech recognition errors [76]. Another aspect of clinical documentation is the coding and classification of examinations or diseases [77]. Furthermore, LLMs can facilitate the translation and summarization of medical information. In our analysis, most studies showed the feasibility of LLMs in processing languages other than English for SR [35–37, 40, 41]. In addition to language translation, LLMs could support language correction/editing, medical text summarization, or text simplification, e.g., when reporting examination results to patients, but also for streamlining imaging protocols [78, 79]. Another important solution that LLMs can offer is the automation of data mining, e.g., for retrieving or extracting medical information to prepare large data sets for research purposes, as recently demonstrated for free-text CT reports on lung cancer [80]. Finally, LLMs offer great opportunities to accelerate clinical evaluation by automating the referral/follow-up depending on the report results, providing differential diagnoses, or supporting the clinical decision process by providing treatment recommendations or prognosis predictions [71, 81–83]. We have schematically summarized the current and prospective applications of LLMs within the scope of radiology report processing in Fig. 2.

Beyond language processing tasks, multimodal generative models are set to redefine image-based diagnostics. While current multimodal generative models such as GPT-4V have demonstrated their ability to identify pathologies in selected images, outperforming GPT-4 without images in several radiological subspecialties, they have yet to match the performance of specialized deep learning models that can analyze the full sequence of images slice by slice [84]. However, with the advent of more advanced capabilities, multimodal generative models hold great potential for image analysis and processing, for example, by informing about incidental findings that were overlooked or improving image reconstruction directly from k-space [85, 86]. Nevertheless, so far, the ability of multimodal generative models to aid in medical imaging diagnosis remains a prospect for the future.

## Conclusions

Our review provides a comprehensive overview of the past, present, and future of LLMs in structured radiology reporting and beyond. The shift towards more sophisticated models for NLP tasks has unlocked new opportunities for SR in radiology. However, the current literature on the use of LLMs for SR is limited in quantity and scope, with most studies focusing on the application of GPT-3.5 and GPT-4. Nevertheless, all studies reported favorable results and acknowledged the potential of LLMs for SR in radiology. Although the impact of LLMs on radiology and the broader medical field could redefine the medical workforce and economy with the advent of more advanced capabilities, overcoming regulatory frameworks, such as introduced by the European Union’s AI Act and the associated challenges for generative AI and LLMs, including untransparent algorithms and training data for proprietary models, will determine the extent to which LLMs can be successfully implemented into SR and clinical practice. Finally, there are still some unanswered questions at this stage that require further investigation. These include, for instance, a comparison of the structured reports generated by LLMs with those produced by radiologists, the optimal integration of LLMs into existing clinical software and systems, and the question of whether reports generated by LLMs are as clinically accepted as those produced by radiologists alone. Future studies should also investigate the user-friendliness of LLM-generated reports for both radiologists and patients, as well as the clinical acceptance and integration of these systems in real-world settings.

## Abbreviations

BERT: Bidirectional encoder representations from transformers
CEUS: Contrast-enhanced ultrasonography
GPT: Generative pre-trained transformer
LLMs: Large language models
LSTM: Long short-term memory networks
NLP: Natural language processing
RIS: Radiology information systems
SR: Structured reporting
